# Supplementary material for: Supercritical Fluid Extraction of Phenolic Compounds from Mango (Mangifera indica L.) Seed Kernels and Their Application as an Antioxidant in an Edible Oil
Source: Molecules. 2021 Dec 11;26(24):7516. doi: 10.3390/molecules26247516 (PMC8703722; doi:10.3390/molecules26247516)
Supplement: Supplementary file 1 [file molecules-26-07516-s001.zip › molecules-1479927-supplementary.pdf]

Supplementary Material

Supercritical Fluid Extraction of Phenolic Compounds from Mango (*Mangifera indica* L.) Seed Kernels and their Application as an Antioxidant in an Edible Oil

Luis Miguel Buelvas-Puello [1], Gabriela Franco-Arnedo [1], Hugo A. Martínez-Correa [2], Diego Ballesteros-Vivas [3], Andrea del Pilar Sánchez-Camargo [4], Diego Miranda-Lasprilla [5], Carlos-Eduardo Narváez-Cuenca [1] and Fabián Parada-Alfonso [1,*]

[1] Food Chemistry Research Group, Departamento de Química, Facultad de Ciencias, Universidad Nacional de Colombia, Sede Bogotá, Carrera 45 No 26-85, Bogotá D.C. 111321, Colombia; lmbuelvasp@unal.edu.co (L.M.B.-P.); gafrancoar@unal.edu.co (G.F.-A.); cenarvaezc@unal.edu.co (C.-E.N.-C.)

[2] Departamento de Ingeniería, Universidad Nacional de Colombia, Sede Palmira, Carrera 32 No. 12-00, Palmira 763531, Colombia; hamartinezco@unal.edu.co

[3] Departamento de Nutrición y Bioquímica, Facultad de Ciencias, Pontificia Universidad Javeriana, Bogotá D.C. 111321, Colombia; diego_ballesteros@javeriana.edu.co

[4] Grupo de Diseño de Productos y Procesos (GDPP), Department of Food and Chemical Engineering, Universidad de los Andes, Carrera 1E No. 19 A 40, Edificio Mario Laserna, Bogotá 111711, Colombia; ad.sanchez@uniandes.edu.co

[5] Facultad de Ciencias Agrarias, Universidad Nacional de Colombia, Sede Bogotá, Carrera 45 No 26-85, Bogotá D.C. 111321, Colombia; dmirandal@unal.edu.co

* Correspondence: fparadaa@unal.edu.co; Tel.: +57-1-3165000 (ext. 14480); Fax: + 57-1-3165220

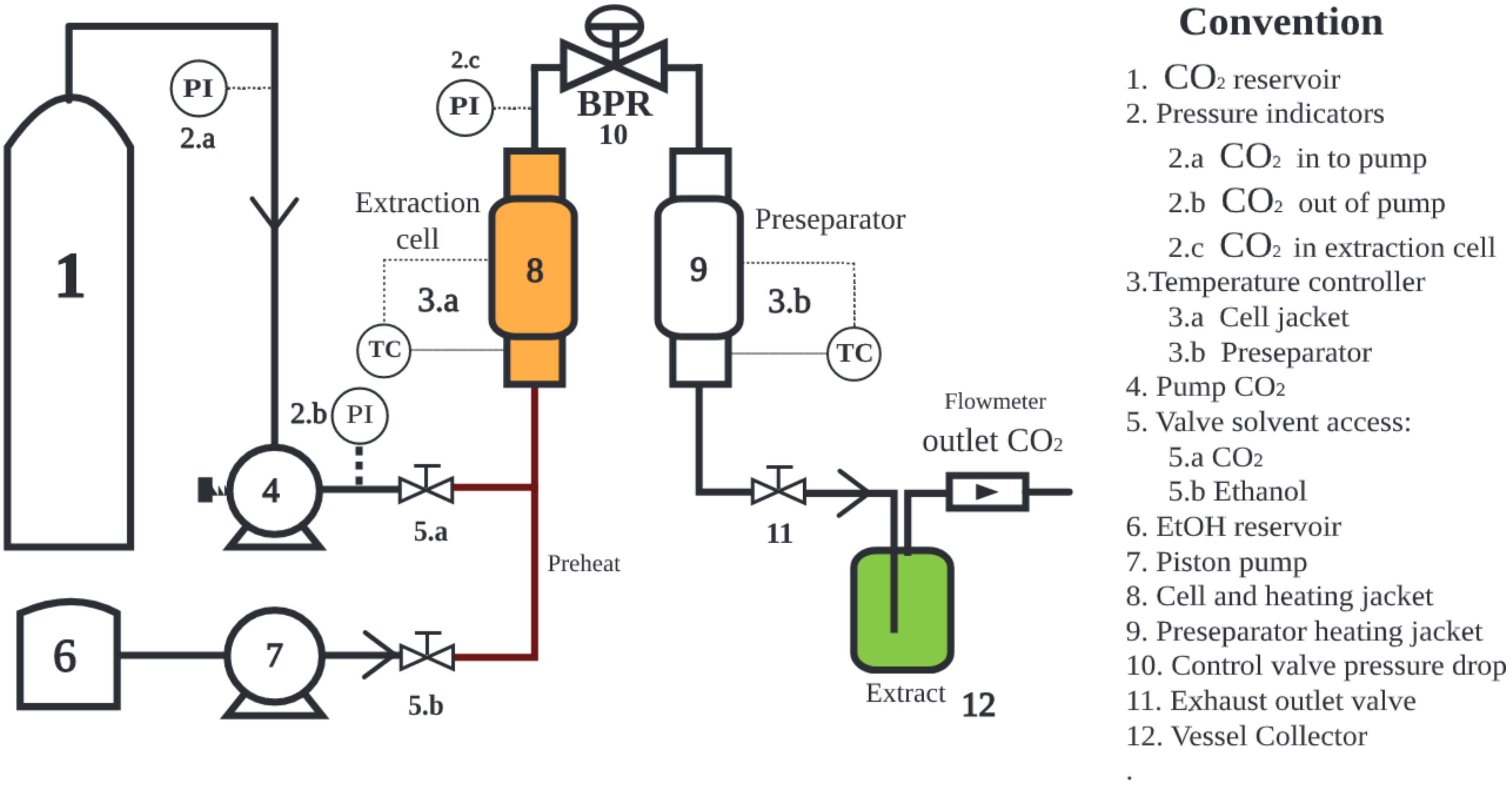

Figure S1. Schematic diagram of the equipment employed for SFE (home-build experimental unit).

Table S1. Retention time, UV-Vis spectra, and quantification of compounds by supercritical fluid extraction as well as by Soxhlet extraction.

Feature	Peak 1[a]	Peak 2[b]	Peak 3	Peak 4	Peak 5	Peak 6	Peak 7	Peak 8
Retention time (min)	2.95	13.53	13.86	14.48	14.68	14.92	15.22	16.34
UV-Vis max (nm)	272	274	357; 368	281	281	280	280	276
**Quantification in supercritical fluid extraction**								
1	37.8	41.3	35.7	70.1	177.8	7.7	55.0	43.3
2	37.7	67.3	10.0	8.8	21.7	6.5	9.7	14.4
3	21.7	19.0	30.6	79.8	126.2	96.4	81.3	20.0
4	44.0	63.8	19.1	5.1	85.1	3.7	23.7	25.6
5	43.8	61.3	8.4	7.5	19.0	6.3	9.4	18.6
6	40.0	20.9	6.9	2.7	22.2	1.9	6.5	9.1
7	28.2	10.5	3.7	2.3	10.4	2.0	6.8	9.3
8	51.1	58.3	10.9	8.6	21.6	6.6	9.8	16.9
9	20.0	18.3	15.7	9.1	134.4	4.2	35.1	36.5
10	59.9	163.8	8.8	3.9	9.2	3.9	5.8	28.2
11	48.4	19.5	19.9	24.6	58.8	3.3	17.1	12.3
12	25.9	66.3	18.2	5.9	81.3	3.6	21.2	18.0
13	48.1	51.3	8.9	7.5	18.6	6.4	9.3	17.3
14	48.3	178.0	7.1	5.6	14.5	5.3	7.6	16.5
15	41.4	92.3	10.5	14.0	35.0	10.8	16.1	21.8
16	25.0	51.4	39.0	44.3	116.6	4.0	36.1	26.5
17	46.6	12.4	28.5	31.2	69.3	4.1	26.5	9.4
18	8.4	10.3	33.2	37.1	86.3	4.7	32.5	7.7
19	40.8	67.0	7.2	7.4	17.8	5.9	8.8	17.6
**Quantification in Soxhlet extraction**								
	11.0	7.1	45.7	79.4	105.8	47.8	68.5	12.9

[a] Retention time and UV-Vis spectra coincident with authentic standard gallic acid. [b] Retention time and UV-Vis spectra coincident with authentic standard ellagic acid. Numbers 1–19 in the first column correspond to the experiments performed by supercritical fluid extraction, following the same numbering as in Table 1. In those measurements no technical replications were performed. In contrast, in Soxhlet extraction three replications were performed. Concentration of peak 1 was expressed as mg gallic acid g^{-1} extract. Concentration of peak 2 was expressed as mg ellagic acid g^{-1} extract. Concentration of peaks 3–8 were expressed as mg ellagic acid equivalents g^{-1} extract.
